# Pituitary macroadenoma presenting as a nasal tumor: case report

**DOI:** 10.1590/1516-3180-2014-1326693

**Published:** 2014-09-02

**Authors:** Nivaldo Adolfo Silva, Fabiano Reis, Larissa Kaori Miura, Guilherme Henrique Vieira, Luciano Souza Queiroz, Heraldo Mendes Garmes, Cristina Laguna Benetti-Pinto

**Affiliations:** I MD, PhD. Attending Physician, Department of Radiology, Faculdade de Ciências Médicas, Universidade Estadual de Campinas (Unicamp), Campinas, São Paulo, Brazil; II MD, PhD. Professor, Department of Radiology, Faculdade de Ciências Médicas, Universidade Estadual de Campinas (Unicamp), Campinas, São Paulo, Brazil; III MD. Resident, Department of Radiology, Faculdade de Ciências Médicas, Universidade Estadual de Campinas (Unicamp), Campinas, São Paulo, Brazil; IV MD. Faculdade de Ciências Médicas, Universidade Estadual de Campinas (Unicamp), Campinas, São Paulo, Brazil; V MD, PhD. Professor, Department of Pathology, Faculdade de Ciências Médicas, Universidade Estadual de Campinas (Unicamp), Campinas, São Paulo, Brazil; VI MD, PhD. Assistant Physician, Department of Endocrinology, Faculdade de Ciências Médicas, Universidade Estadual de Campinas (Unicamp), Campinas, São Paulo, Brazil; VII MD, PhD. Professor, Department of Obstetrics and Gynecology, Faculdade de Ciências Médicas, Universidade Estadual de Campinas (Unicamp), Campinas, São Paulo, Brazil

**Keywords:** Pituitary neoplasms., Nasal cavity, Growth hormone-secreting pituitary adenoma, Amenorrhea, Prolactin, Neoplasias hipofisárias, Cavidade nasal, Adenoma hipofisário secretor de hormônio do crescimento, Amenorreia, Prolactina

## Abstract

**CONTEXT:**

Pituitary macroadenomas are rare intracranial tumors. In a few cases, they may present aggressive behavior and invade the sphenoid sinus and nasal cavity, causing unusual symptoms. In this paper, we report an atypical case of pituitary adenoma presenting as a nasal mass.

**CASE REPORT:**

The patient was a 44-year-old woman who had had amenorrhea and galactorrhea for ten months, with associated nasal obstruction, macroglossia and acromegaly. Both growth hormone and prolactin levels were increased. Magnetic resonance imaging showed a large mass originating from the lower surface of the pituitary gland, associated with sella turcica erosion and tumor extension through the sphenoid sinus and nasal cavity. Histopathological analysis demonstrated a chromophobe pituitary adenoma with densely packed rounded epithelial cells, with some atypias and rare mitotic figures. There was no evidence of metastases.

**CONCLUSION:**

Macroadenoma invading the nasal cavity is a rare condition and few similar cases have been reported in the literature. This study contributes towards showing that tumor extension to the sphenoid sinus and nasopharynx needs to be considered and investigated in order to make an early diagnosis when atypical symptoms like nasal obstruction are present.

## Abstract (pt)

**CONTEXTO:**

Macroadenomas hipofisários são tumores intracraniais raros. Em alguns casos, podem apresentar comportamento agressivo e invadir o seio esfenoidal e a cavidade nasal, causando sintomas não usuais. Neste relato de caso, descrevemos um caso atípico de adenoma hipofisário manifestando-se como uma massa nasal.

**RELATO DE CASO:**

A paciente de 44 anos, do sexo feminino, apresentava amenorreia e galactorreia por 10 meses associando-se a obstrução nasal, macroglossia e acromegalia. Os níveis do hormônio de crescimento e de prolactina apresentaram-se aumentados. Ressonância magnética mostrou uma grande massa originada da superfície inferior da glândula hipofisária associada com erosão da sela túrcica e extensão do tumor através do seio esfenoidal e cavidade nasal. Análise histopatológica demonstrou adenoma hipofisário cromófobo com células epiteliais arrendondadas densamente agrupadas com algumas atipias e escassas figuras de mitose. Não houve evidências de metástase.

**CONCLUSÃO:**

O macroadenoma invasivo para a cavidade nasal é uma condição rara e há poucos relatos similares descritos na literatura. Este trabalho contribui para mostrar que, na presença de sintomas atípicos como a obstrução nasal, a extensão para o seio esfenoidal e para a nasofaringe deve ser considerada e investigada para um diagnóstico precoce.

## INTRODUCTION

Pituitary macroadenomas are rare intracranial tumors. They may occur in different settings of presentation and measure more than 10 mm in diameter. Usually, they exhibit endocrine symptoms and local mass effects that may include compression of the optical chiasm and consequent visual disturbance. In some cases, they become invasive, going beyond the sella turcica to adjacent structures such as the internal carotid and cavernous sinus. In rare cases, they extend to the sphenoid sinus and nasopharynx.^1^^-^ 3 When this occurs, some unusual symptoms may appear, which may or may not be accompanied by the expected findings, like epistaxis, nasal obstruction, painful sinuses and purulent rhinorrhea.^4^^-^ 8

The reason why certain adenomas behave in a locally aggressive fashion is unknown and, although most cases are benign, these tumors can sometimes show malignant characteristics and so can be defined as carcinomas in the presence of craniospinal and/or systemic metastases.^9^^,^ 10 In the present case, scintigraphy and computed tomography ruled out the diagnosis of a malignant tumor. The incidence of pituitary carcinoma is relatively low in patients with invasive macroadenomas and, in many cases, the metastases may be silent.^6^ Since this is an important differential diagnose for other tumors involving the skull base, correct investigation is indispensable, given that pituitary neoplasms have a much more favorable prognosis and low metastatic potential.^4^^,^ 10

Furthermore, improvements in diagnostic methods such as magnetic resonance imaging techniques and pathological examinations including immunohistochemical and ultrastructural studies provide the possibilities of precisely delimiting the extent of the mass and describing the composition and kind of tumor.^11^ In this paper, we report a rare case of pituitary macroadenoma that was found to present growth extending through the sella turcica to invade the sphenoid sinus and nasopharynx. Although this case represents an unusual kind of presentation, we show that invasive adenomas may manifest primarily as a nasal mass.

## CASE REPORT

A 44-year-old woman who had had amenorrhea and galactor rhea for 10 months, had been noting progressive growth of her face and extremities over the past two and a half years. Over the last six months, she had complained of severe nasal obstruction with respiratory distress and dysphagia due to macroglossia. After referral to our service, which forms part of a tertiary-level uni versity hospital belonging to Universidade Estadual de Campinas (UNICAMP), she started to be followed up regarding endocrinol ogy, neurosurgery and ophthalmology. 

**Figure 1:**
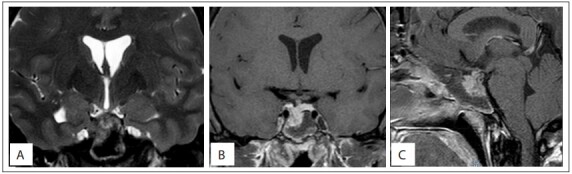
Magnetic resonance imaging with coronal T2 and T1 fat saturation post-gadolinium weighting, showing a lesion originating from the lower right surface of the hypophysis associated with sella turcica erosion and extending through the sphenoid sinus (A, B). Magnetic resonance imaging with sagittal T1 fat saturation post-gadolinium weighting, showing the extension through the nasal cavity (C).

On physical examination, acromegaly was evident. The blood hormone levels were: growth hormone (GH) 34.20 ng/ml (n = 0.06-5.0 ng/ml), insulin-like growth factor-1 (IGF-1) 726 ng/ml and prolactin (PRL) ranging from 70 to 82.83 ng/ml in three samples (n = 4.79 - 23.3 ng/ml). ACTH, TSH, FSH and LH levels were all normal. Magnetic resonance imaging (MRI) depicted a large mass (3.2 x 4.1 x 3.2 cm) originating from the lower surface of the pituitary gland, which was associated with sella turcica erosion and tumor extension through the sphenoid sinus into the nasal cavity (Figure 1). The sella itself was of normal dimensions and, because the tumor had expanded on the lower side, the optical chiasm was not affected, as was verified through a normal visual field examination. 

Initially, considering the high levels of GH and that, besides surgery, adjuvant therapy would probably be necessary in order to reduce the tumor, pharmacological treatment with octreotide 30 mg (a somatostatin analogue that inhibits GH and IGF-1) was administered every 28 days. However, after three months, the blood hormone levels were seen to remain elevated and the symptoms became severe. Therefore, endoscopic surgery was indicated. 

The surgical procedure was started by setting the speculum at the nostril, so as to reach the tumor through the transsphenoidal route. Because of the volume of the intranasal mass, the procedure was halted due to technical difficulties and only a biopsy was performed. 

Two months later, it was decided to perform transcranial surgery. Through bifrontal craniotomy, the anterior cranial fossa was accessed and, after drilling the sphenoid surface, the mass was aspirated and removed. 

**Figure 2:**
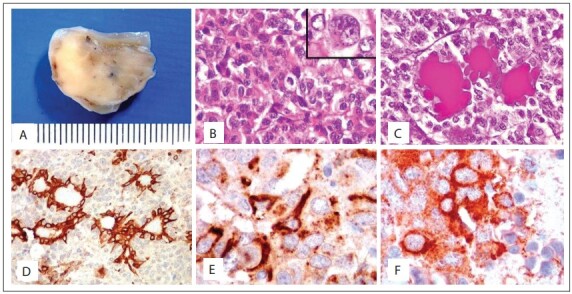
Polyp-like specimen excised from left nasal cavity. External surface was smooth and shiny, cut surface solid and firm, and translucent in some areas (A). Chromophobe pituitary adenoma showing densely packed rounded epithelial cells with some atypia and scattered mitotic figures (one at the center) (hematoxylin-eosin, HE; 100 X); inset: binucleated cell with prominent nucleoli and rare cytoplasm (400 X) (B). Glands of nasal mucosa surrounded by tumor cells, in which mucus is highlighted in magenta in the dilated gland lumina (periodic-acid Schiff, PAS; 100 X) (C). Immunohistochemical analysis for 34Βe12 (an antibody for high molecular weight keratins), demonstrating mucosal glands trapped amid negative adenoma cells (100 X) (D). Immunohistochemical analysis (400 X) of the pituitary adenoma cells in the cytoplasm, which are focally positive for growth hormone (GH) (E) and prolactin (F).

Histopathological analysis on the biopsy and the fragments from the hypophysectomy gave rise to a diagnosis of infiltrating pituitary macroadenoma with nuclear atypia. Immunohistochemistry on the samples showed that they were positive for GH and PRL (Figure 2). A visual estimate of marked nucleus proportions showed that 3-5% were positive for Ki-67, with several typical mitoses, thus characterizing an atypical pituitary tumor. Since the diagnosis of carcinoma is confirmed only if metastases are present, whole-body bone scintigraphy and abdominal, pelvic and chest computed tomography were performed. There was no evidence of metastases.^12^

**Figure 3:**
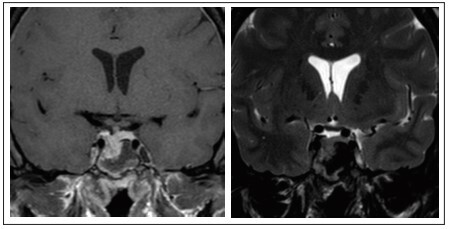
Magnetic resonance imaging with coronal T1 fat saturation post-gadolinium and T2 weighting, two months after surgery, showing tumor residues on the lower right surface of the hypophysis.

Two months after the surgery, the patient reported that her menstrual cycles had become regular and that her galactorrhea had ceased. Three months later, she noticed that her shoe size had decreased by two sizes. Although her GH and PRL hormone levels showed progressive reduction over this period (GH = 3.41 ng/ml; PRL= 12.44 ng/ml), IGF-1 maintained high levels (IGF-1 = 455 ng/ml). A postoperative control MRI examination showed evidence of tumor residues (Figure 3). The patient continues to be asymptomatic, with continuing pharmacological treatment with octreotide and endocrinological follow-up.

## DISCUSSION

Even though pituitary macroadenoma is a rare condition, it exhib its a variety of presentations and manifestations of symptoms when invading the sphenoid sinus and nasal cavity. In the present case, the appearance of some typical signs such as amenorrhea, galactor rhea and growth of the face, tongue and extremities (resulting from increased GH and prolactin levels) contributed towards the inves tigation and aided in making the diagnosis. 

However, in many cases reported in the literature, less exuberance of symptoms was reported. The patients showed nasal obstruction, sometimes with epistaxis and purulent rhinorrhea. In addition, the lack of endocrinal symptoms and the normal levels of pituitary hormones made it more difficult to suspect an adenoma, with a consequent risk of diagnostic delay. In almost all of the cases, the diagnosis was only made after removing the obstructing tissue for microscopic examination.^6^^,^ 7

Invasive pituitary neoplasm may occur either as an extension from an intrasellar lesion or as an ectopic tumor. Kikuchi et al. and other authors described similar cases of a large pituitary adenoma consequent to an ectopic focus in the nasal cavity.^13^ In the present case, the histological analysis and the imaging examinations reinforced the evidence showing that the pituitary macroadenoma extended inside the sella turcica. Magnetic resonance imaging showed that the mass originated from the right lower surface of the pituitary gland, with associated sella turcica erosion and tumor extension through the sphenoid sinus into the nasopharyx.^14^ However, when the tumor originates from an ectopic focus, with a normal pituitary gland, it becomes more difficult to restrict the differential diagnoses. 

Although pituitary adenomas are considered to be benign tumors, they can show malignant characteristics when the presentations are atypical, such as in the case reported here. Invasive pituitary tumors are relatively rare, as are pituitary carcinomas. In cases of the latter condition, a pituitary tumor that is either not contiguous with the primary sellar tumor and/or a pituitary tumor that has metastasized to distant sites from the pituitary gland needs to be present.^12^ This shows that for correct diagnosis in cases of tumors in the nasal cavity, it is very important to exclude other intracranial tumors, such as craniopharyngioma, chordoma, chondrosarcoma and meningioma.^9^ This diagnosis is usually made by means of histological analysis, using the endocrine growth pattern comprising tumor cells arranged in packets, ribbons or rosettes, with delicate neuroendocrine markers and pituitary hormones.

It seems that invasive pituitary macroadenomas do not follow any standard. As shown in Table 1, few cases have been described in the literature and there is still much to study about these tumors, given that the reason why some of them behave in a more locally aggressive manner remains unknown.^10^ The case presented here is important, because it shows that even when the presentation is atypical, with only a few symptoms like nasal obstruction, the diagnostic hypothesis of macroadenoma needs to be taken into consideration whether or not its usual clinical signs are present, especially when caused by variation in endocrinal hormone levels.

**Table 1:** Search strategies performed on June 25, 2013, and the results from each database

Electronic databases	Key words	Results	
		Found	Related
Medline via PubMed	(pituitary adenomas) AND (nasal cavity) AND (obstruction)	12	5
Lilacs via Bireme	(pituitary neoplasms) OR (neoplasias hipofisarias) OR (neoplasias hipofisárias) AND (sphenoid sinus) OR (seno esfenoidal) OR (seio esfenoidal) AND (nasal obstruction) OR (obstrucción nasal) OR (obstrução nasal)	18	0
Embase via Elsevier	(pituitary adenomas) AND (nasal cavity) AND (obstruction)	0	0

## CONCLUSION

Invasive macroadenoma to the nasal cavity is a rare condition with few cases reported in the literature and the reason why some are more locally aggressive is still unknown. The description of this case of atypical presentation and its peculiarities shows that exten sion of the tumor to the sphenoid sinus and nasopharynx needs to be taken into consideration when the usual hormonal manifes tations of adenomas present associations with nasal symptoms. In this manner, early diagnosis and intervention may be facilitated. 
